# Prediction of postoperative nausea and vomiting by point-of-care gastric ultrasound: can we improve complications and length of stay in emergency surgery? A cohort study

**DOI:** 10.1186/s12871-021-01428-0

**Published:** 2021-08-31

**Authors:** Valerio Cozza, Lorenzo Barberis, Gaia Altieri, Mario Donatelli, Gabriele Sganga, Antonio La Greca

**Affiliations:** 1 Fondazione Policlinico Universitario A. Gemelli IRCCS, Roma, Italy; 2 Università Cattolica del Sacro Cuore, Roma, Italy; 3 Emergency Surgery and Trauma, Fondazione Policlinico Universitario A. Gemelli IRCCS - Università Cattolica del Sacro Cuore, Roma, Italy

**Keywords:** Postoperative nausea and vomiting, Postoperative ileus, Gastric ultrasound, Gastric emptying, Acute care surgery

## Abstract

**Background:**

Postoperative nausea and vomiting and postoperative ileus are common after major digestive surgery and represent one of the significant problems in Acute Care Surgery.

The delivery model of emergency surgery needs to be improved in order to foster a patient-centered care.

The multimodal approach suggested by Enhanced Recovery After Surgery (ERAS®) Guidelines is gaining widespread acceptance but is difficult to apply to emergency surgery.

Ultrasound examination of the gastric antrum allows a reliable assessment of gastric contents and volume and might help contribute to improve perioperative care in the emergency setting.

**Methods:**

Gastric ultrasound examinations were performed preoperatively and postoperatively on forty-one patients undergoing emergency abdominal surgery. Gastric cross-sectional area (CSA) was measured, in order to estimate the gastric volume. The data obtained were used to evaluate a possible relationship between delayed gastric emptying and postoperative adverse event.

**Results:**

Gastric antrum detection rate varied from 31.8% in open up to 78.9% in laparoscopic surgeries (*p* = 0.003). Six patients experienced adverse outcomes, had an antiemetic therapy administered and/or a nasogastric tube inserted. Mean CSA was significantly higher in this group (12.95 cm^2^ vs 6.12 cm^2^; *p* = 0.040).

**Conclusions:**

Sensitivity of gastric ultrasound varies depending on surgical technique. A dilated gastric antrum is significantly related to postoperative adverse outcomes and a careful ultrasound follow-up might help tailor postoperative nutrition and antiemetic therapy. In patients who experienced adverse events, antral CSA showed an average increase of more than 50% over a period of 72 h after surgery. A relative measure could be used to predict the risk of postoperative ileus. Overall, gastric ultrasound seems to be a promising diagnostic tool and a useful way to integrate ERAS® protocol in emergency abdominal surgery.

## Background

Lack of bowel function, postoperative nausea and vomiting (PONV) and abdominal distension are common morbidities in the early postoperative period, especially in patients who underwent major abdominal surgery. In some patients, this dysfunction may be prolonged, resulting in postoperative ileus (POI) [1].

Currently, the diagnosis of POI is mostly based on clinical symptoms and/or the need for a nasogastric tube (NGT). Current recommendations suggest that the delivery model of Emergency Surgery (ES) needs to be changed in order to improve efficiency, quality of care and decrease overall mortality [2]. The multimodal approach suggested by Enhanced Recovery After Surgery (ERAS®) Guidelines [3] in order to ensure a safe methodology for minimising the negative impact of surgery on organ function and particularly on preventing PONV and POI is gaining widespread acceptance but is difficult to apply to ES.

POI exerts a significant impact on Enhanced Recovery After Surgery (ERAS®) programs, because it is a frequent reason for delayed recovery and prolonged hospital stay as well as the cause of highly feared complications such as pulmonary aspiration (PA).

As reported in the ERAS® Society guidelines [3], in order to prevent POI, a multimodal approach can be adopted (e.g. laparoscopic technique, avoidance of routine NGT, opioid-sparing analgesia).

Unfortunately, in the emergency setting, a preventive approach according to ERAS® guidelines is only rarely possible. For these patients, NGT is frequently used as a diagnostic tool and routine intervention to decompress the stomach when ileus is just suspected, in order to prevent PA.

Nowadays, multiple studies have proven that postoperative prophylactic NGTs are not beneficial [4].

Antiemetic prophylaxis is reserved to patients at high risk of PONV according to the Apfel score [5]. However, such a score takes into account only clinical parameters and is not accurately predictive of PONV.

As a matter of fact, ERAS® guidelines have been also applied to emergency abdominal surgery, with heterogeneous results [6–8]. In particular, a multimodal preventive approach to events like PONV and POI, limitation in the use of NGT, and early oral feeding have been demonstrated difficult to apply in ES.

Therefore, a non-invasive and largely available technique is needed to detect those patients undergoing emergency surgery who would take advantage of a NGT insertion and those who, instead, can be addressed safely towards an enteral feeding and an early recovery. This technique should offer a measurable, reproducible and standardized tool to be easily applied to all patients at the bedside.

Routine ultrasound (US) assessment of the stomach, performed both preoperatively and postoperatively, might meet these requirements and help distinguish those patients whose PONV is sustained by an abnormal gastric pool and/or gastric emptying rate (GER). Thus, gastric ultrasound (GUS) results may help to reduce NGT placement, select appropriate antiemetic drugs with the most appropriate mechanism of action, tailor postoperative hydration and feeding, and possibly allow faster and uneventful recovery, according to the principles of ERAS® protocol.

Ultrasound assessment of the stomach received a great deal of attention in the past years, thanks to its proved efficacy in determining the type of gastric content and volume in healthy subjects [9, 10], in surgical patients [9, 11], in pregnant women [12–14], in end stage renal failure individuals [15], after bowel preparation prior to colonoscopy [16], after ingestion of carbohydrate-rich meals [17] and in individuals with altered gastric motility [18]. At the moment, the majority of data on ultrasound assessment of the stomach derive from experimental studies, but also from clinical studies correlating the risk of aspiration prior to anesthesia and the effects of different types of nutrition or medications on gastric emptying [19].

Gastric ultrasound (GUS) has been studied extensively in the anesthesiology field. A prospective observational trial by Bouvet et al. [20, 21] proved a correlation between the preoperative ultrasound-assessed antral cross-sectional area (CSA) and the volume of aspirated gastric contents. Perlas et al. [10] found that antral CSA correlated with volumes of up to 300 mL in a close-to-linear fashion, particularly when subjects were in the right lateral decubitus (RLD) position. Moreover, sonographic assessment of the gastric antrum provides qualitative information about gastric content (empty or not empty) and its nature (gas, fluid, or solid). In 2014, A. Perlas and P. Van de Putte published a systematic review [22] that collects the state-of-the-art on this technique, how it should be carried out, and finally proposed a flowchart that would allow to predict the risk of aspiration perioperatively. At the same time, a thorough assessment of feasibility, accuracy and affordability of gastric ultrasound in the emergency setting is still lacking.

## Materials and methods

### Aim of the study

The primary aim of this study is to assess the feasibility of bedside sonographic evaluation of the stomach in patients undergoing emergency abdominal surgery, in order to predict the risk of PONV using Gastroesophageal reflux disease (GERD)-related parameters.

The secondary aim is to match the quantitative and qualitative measurements of gastric antrum to the clinical status, gastrointestinal (GI) function and actual postoperative course of patients (e.g. placement of NGT, administration of antiemetics, PONV), retrospectively.

### Study design

This is a single center prospective cohort study, conducted over a period of 6 months, from January 2019 to June 2019.

The reporting of this study conforms to the STROBE statement [23].

The study population included forty-one patients admitted to the division of Emergency Surgery and Trauma of our hospital, undergoing urgent abdominal surgery (i.e. surgery that cannot be postponed for more than 48 h since clinical onset).

All the patients were managed according to the principles of the ERAS® protocol [3].

Subjects were recruited in the Emergency Department and in the Emergency Surgery ward after being found eligible for surgical intervention, carried out as an urgent procedure, by the surgical team of this unit.

GUS was performed multiple times during the hospitalization, once preoperatively, and 3 to 5 times during the postoperative period. The preoperative exam was carried out after at least a 6-h fasting period.

The following clinical parameters were recorded through daily questioning of patients and review of patients’ notes:

- Daily tolerated diet: solid/semiliquid/liquid
- Daily fluid intake
- Recovery of peristalsis (defined as passage of flatus or feces)
- Nausea and/or vomiting
- Abdominal distention
- NGT placement
- Abdominal pain
- Analgesia and antiemetic therapy requirement.

### Gastric antrum detection

**Fig. 1:**
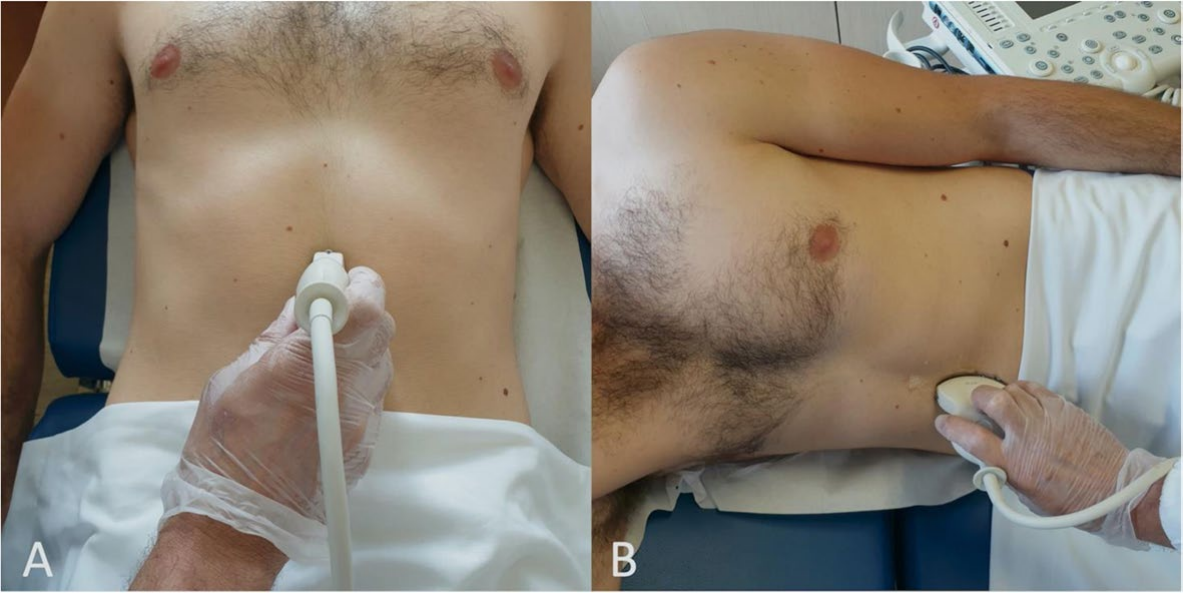
Scanning technique. Two scans were performed, one in supine (**A**) and another one in right lateral decubitus (**B**). In both positions, the gastric antrum was detected by positioning the probe on the epigastric region, on the sagittal and parasagittal plane

All US examinations were carried out by a single operator. An Esaote MyLab^TM^Gamma bedside portable US system was used with a curvilinear array 1- to 8-MHz probe. Two scans were performed, one in semi-recumbent position (with the torso at an angle of 45°) and another one in RLD (Fig. 1). In both positions, the gastric antrum was detected by positioning the probe on the epigastric region, on the sagittal and parasagittal plane. Two important landmarks were used to localize the target portion of the stomach, i.e. the antrum: the left hepatic lobe anteriorly and the abdominal aorta (and the pancreas, if possible) posteriorly [10, 20, 21].

### Qualitative evaluation

**Fig. 2:**
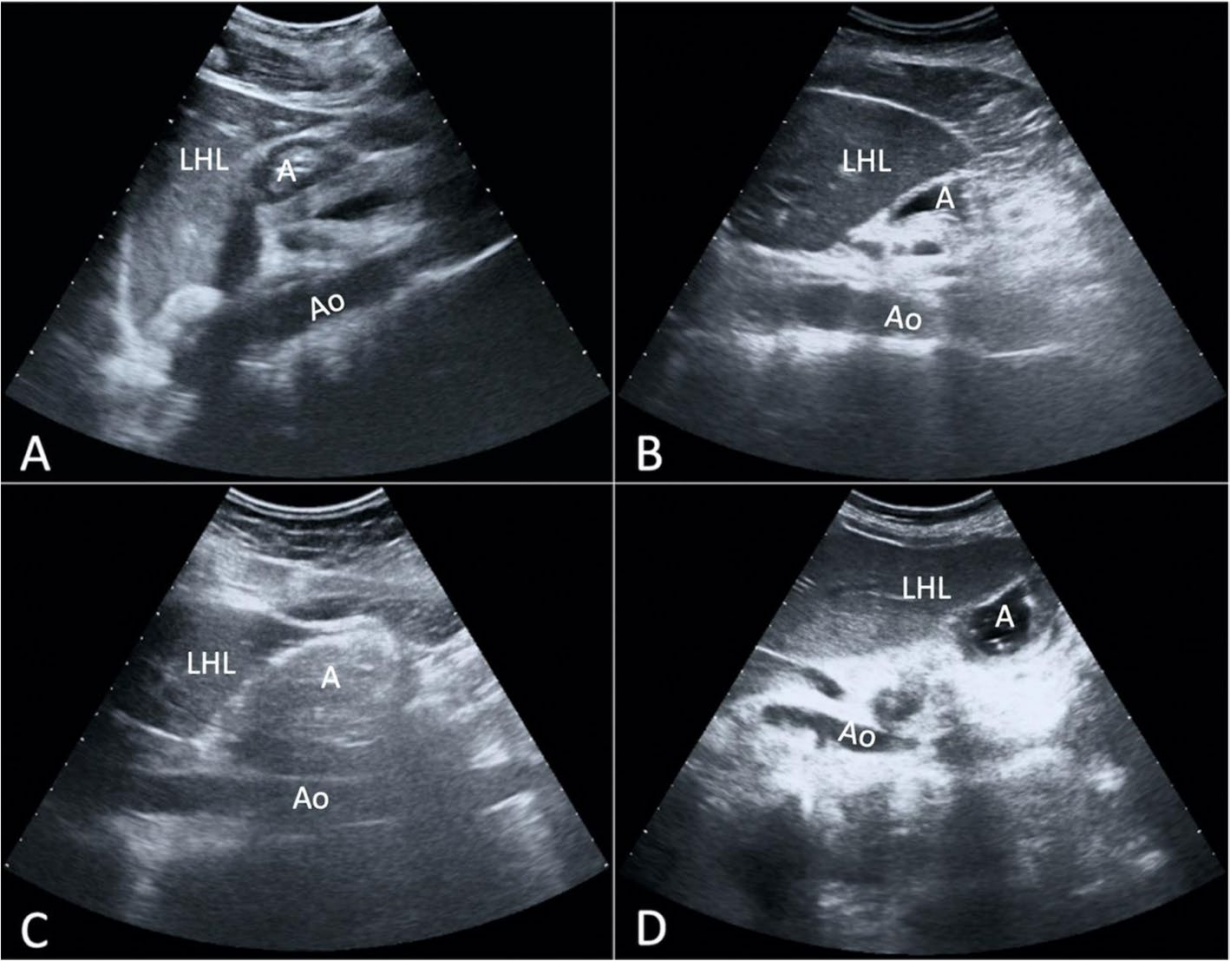
Qualitative evaluation based on the type of gastric contents, reported as empty (**A**); liquid (**B**); solid (**C**); mixed (**D**). Note the different echogenicity of the gastric lumen. A = gastric antrum; Ao = aorta; LHL = left hepatic lobe

A qualitative evaluation was carried out, based on the type of gastric contents; gastric content was described and classified into empty, liquid, solid, or mixed (Fig. 2). The grading system by Perlas et al. [21] was then used, based on the presence of fluid in the gastric lumen in different positions (i.e. Gastric Antral Grade):

- Grade 0 = absence of fluid content both in supine and right lateral decubitus positions;
- Grade 1 = presence of fluid only in right lateral decubitus;
- Grade 2 = presence of fluid both in supine and right lateral decubitus positions.

### Quantitative evaluation

**Fig. 3:**
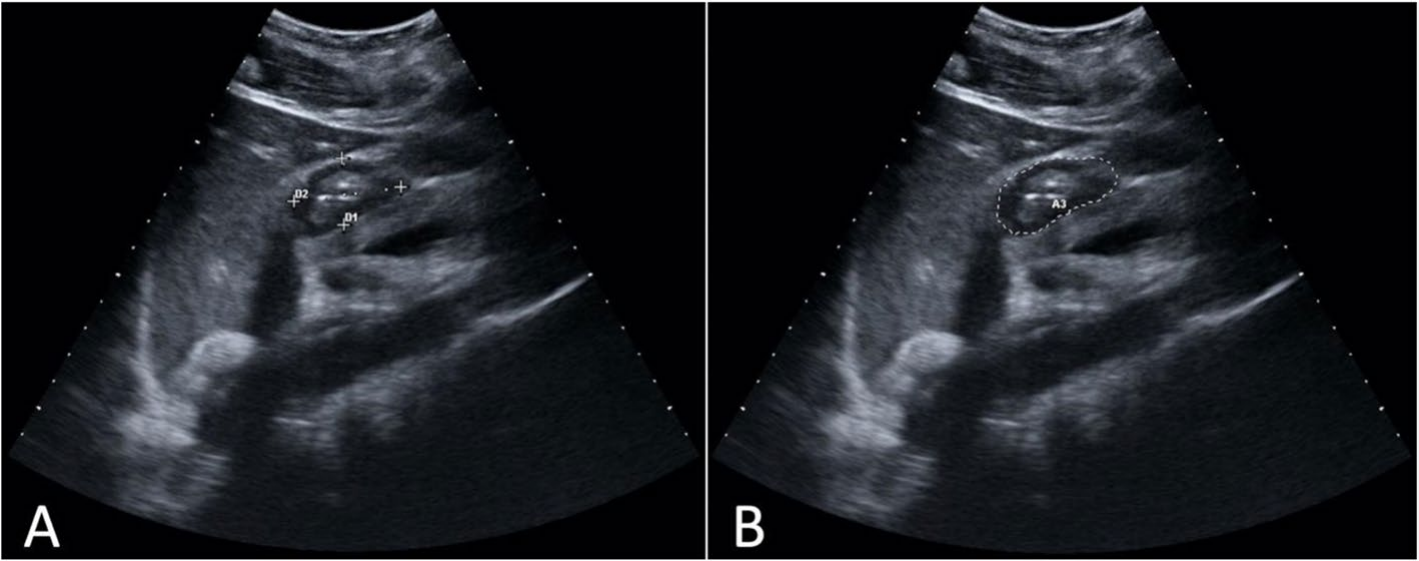
Two methods to measure the antral cross-sectional area: **A** indirectly, through the measurement of two perpendicular diameters (i.e. anteroposterior and cranio-caudal diameters), which are then used to calculate the area of an ellipse with a mathematical formula; **B** directly, through the US machine tracing system

US scans for quantitative assessment were taken during the time interval between two peristaltic contractions and included the entire gastric wall thickness (i.e. serosa-to-serosa) in the measurement. This assessment was carried out via measurement of the Antral Cross-Sectional Area (CSA). This was calculated with two modalities: indirectly, through the measurement of 2 perpendicular diameters (i.e. anteroposterior and cranio-caudal diameters), which were then used to calculate the area of an ellipse with a specific formula (Eq. 1); directly, through the US machine tracing system (Fig. 3). Both measurements were reported using a cm^2^ unit. The CSA measured in such way was then used to calculate the predicted gastric volume, by using the predictive model by Perlas [21] (Eq. 2).

#### Antral cross-sectional area

1 $$CSA\; \left(cm2\right)=Anteroposterior\;diameter*Craniocaudal\;diameter*\pi /4$$

#### Predicted gastric volume

2 $$Volume\;\left(mL\right)=27.0+14.6*RLD\;CSA-1.28*Age$$

### Post hoc subgroup analysis and risk calculation

**Fig. 4:**
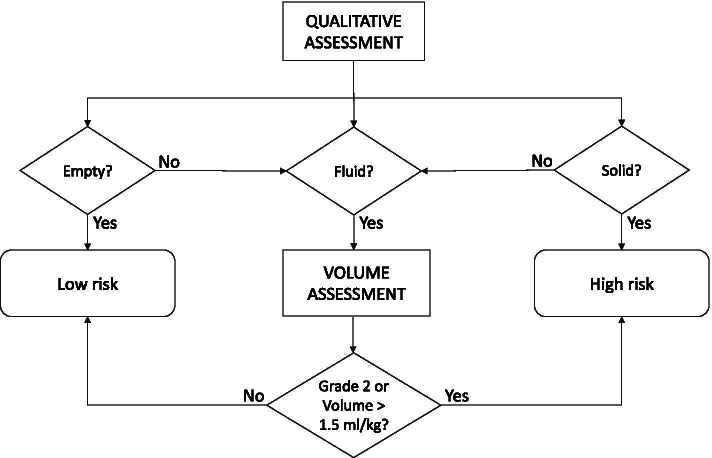
Flowchart for prediction of aspiration risk based on type of gastric content and calculation of gastric volume. This model was applied on this study population in order to assess its feasibility for the purpose of predicting adverse postoperative outcomes in the urgent surgical setting. This algorithm allowed to sort patients out as being at low risk or high risk

A subgroup of 18 patients who underwent major abdominal surgery, including operations entailing a high risk of POI (i.e. total and partial colectomy, small bowel resection) was finally selected. The qualitative and quantitative assessments were integrated using the protocol proposed by Perlas [21] (Fig. 4), in order to assess the applicability of this model in the urgent abdominal surgery setting. The calculated risk was eventually used to correlate the adverse postoperative events occurred in the enrolled subjects, retrospectively.

### Primary outcomes

- Gastric antrum detection rate (%)
- Gastric Antral Grade and type of gastric contents
- Mean, median, range and standard deviation of antral CSA (cm^2^)

### Secondary outcomes

- Number of patients experiencing adverse postoperative events
- Prediction rate of adverse postoperative events (%), based on the algorithm by P. Van de Putte and A. Perlas [21]
- Variation rates (%) of antral CSA (cm^2^) of patients experiencing PONV, over time

### Statistical analysis

Given the innovative nature of the study and the lack of similar studies in the current literature, it was not possible to calculate the sample size. Rather, the number of cases available in the area during the study period determined our study sample size, as well as the achievement of statistical significance.

Categorical variables were expressed as relative and/or absolute frequencies, as well as percentage values. Numerical variables were expressed in terms of median and interquartile range. A Chi-squared test was used to compare categorical variables, while a two-tailed Student T-test was used to compare numerical variables. Finally, a linear regression model was used in order to analyze the relative variation of antral CSA over time. The statistical tests results were reported as p values, and a *p* < 0.05 was considered to be an indicator of a statistically significant difference between selected groups, on two-tailed tests.

## Results

### General characteristics of the study population

Forty-one patients were enrolled and a total of 94 US scans (28/94 performed preoperatively and 66/94 postoperatively) were performed. These examinations spanned different periods of hospitalization: 4/41 (10%) patients were assessed during the preoperative phase only, 12/41 (29%) during the postoperative phase only, 25/41 (61%) during both phases.

The mean age of the sample was 62.2 years, with 19/41 (46.3%) patients younger than 65 years and 22/41 (53.7%) patients with 65 years or above; 13/41 (31.7%) were female and 28/41 (68.3%) were male. Mean BMI was 24.1 kg/m^2^, with 21/41 (51.2%) patients below 25 kg/m^2^ and 20/21 (48.8%) equal to or above 25 kg/m^2^.

The enrolled subjects underwent different types of surgical operations: 12/41 (29.3%) underwent colorectal surgery, 7/41 (17.1%) small bowel surgery, 7/41 (17.1%) cholecystectomy, 7/41 (17.1%) appendectomy, 4/41 (9.8%) hernioplasty, 4/41 (9.8%) other types of operations. Fifteen out of 41 (36.6%) patients had history of previous abdominal surgeries.

### Gastric antrum detection

Gastric antrum was detected in at least one position among supine and Right Lateral Decubitus (RLD) in 62/94 (66%) US scans, while it was not possible to detect it at all in 32/94 (34%) cases.

As concerns gastric US accuracy, there was no statistically significant difference between patient positions, with a detection rate of 59/94 (62.8%) in supine position and 57/94 (60.6%) in RLD (*p* = 0.764). There was no significant difference in antral detection rate among different times of evaluation as well: the antrum was detected in 16/28 (57.1%) preoperative US scans and in 46/66 (69.7%) postoperative US scans (*p* = 0.240).

On the other hand, the type of surgical access had an impact on US accuracy and gastric antrum detection rate varied from 15/19 (78.9%) in laparoscopic to 7/22 (31.8%) in open surgeries (*p* = 0.003).

### Qualitative evaluation

The qualitative evaluation comprised the recording and classification of different types of gastric contents, which were then used to calculate the Gastric Antral Grade by Perlas [21].

Concerning the type of gastric contents in supine position, 40/94 (43%) US scans showed an empty pattern, 10/94 (11%) a liquid one, 3/94 (3%) mixed, 2/94 (2%) solid, and in 39/94 (41%) content could not be recognized. In RLD, 38/94 (40%) showed an empty pattern, 12/94 (13%) a liquid one, 4/94 (4%) mixed, 1/94 (1%) solid, and in 39/94 (41%) content could not be recognized.

Using the data collected on the type of gastric contents, the Gastric Antral Grading was calculated: 34/94 (36.2%) scans had a *G* = *0*, 5/94 (5.3%) had a *G* = *1*, 16/94 (17%) had a *G* = *2* and in 39/94 (41.5%) it could not be calculated.

### Quantitative evaluation

The quantitative assessment of the gastric antrum entailed the measurement of two perpendicular diameters, anteroposterior and craniocaudal, which were then used to calculate the antral CSA indirectly, which is reported here as “CSA_calculated_”. Such measurement was carried out directly as well, with the aid of the US machine tracing system. These measures were reported as “CSA_measured_”. Both CSA_calculated_ and CSA_measured_ were collected in supine and RLD positions. Descriptive statistics were then run on the data sample (Table 1).

**Table 1:** Quantitative measures descriptive statistics

	***Median***	***IQR***
***Preoperative***		
*** D1 (mm)***	24,1	7,8
*** D2 (mm)***	26,1	8,3
*** CSA (cm***^***2***^***) ***_***calculated***_	521,2	267,1
*** CSA (cm***^***2***^***) ***_***measured***_	5,4	3,1
***Postoperative***		
*** D1 (mm)***	23,2	7,9
*** D2 (mm)***	28	10,7
*** CSA (cm***^***2***^***) ***_***calculated***_	505,2	288,3
*** CSA (cm***^***2***^***) ***_***measured***_	5,2	2,4

*SD* Standard deviation, *D1* Diameter 1, *D2* Diameter 2, *CSA* Cross-sectional area, *IQR* InterQuartile Range

Statistical tests were finally performed on CSA_calculated_ and CSA_measured_ in both positions, in order to compare the preoperative and postoperative measurements. The test results came out significant only for the RLD measurements: mean CSA_calculated_ and CSA_measured_ were 4.93 cm^2^ and 5.63 cm^2^ before surgery and 6.92 cm^2^ and 7.25 cm^2^ after surgery (*p* = 0.002 and 0.03, respectively). This means that the gastric antrum is significantly dilated after a surgical operation.

### Subgroup analysis and risk calculation

**Fig. 5:**
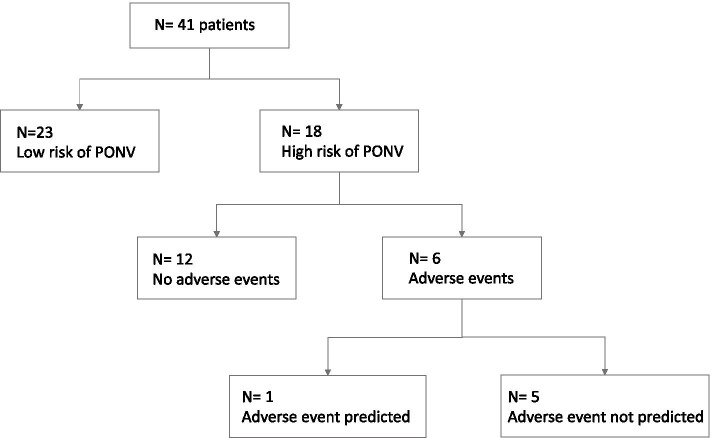
Flowchart showing the subgroup analysis carried out on patients with a higher predicted risk of PONV, based on the type of surgical operation they underwent. PONV = post-operative nausea and vomiting

A subgroup of 18/41 patients who underwent major abdominal surgery was selected, including operations entailing a high risk of POI (i.e. total and partial colectomy, small bowel resection), during which extensive bowel manipulation took place (Fig. 5).

Detection rate in this population was 12/18 (66%).

Six out of 18 patients (33%) experienced episodes of nausea and/or vomiting, had antiemetic therapy administered, had a delayed return of bowel function and/or had an NGT placed. The gastric antrum could be detected and measured in 6/6 (100%) patients who experienced the adverse outcomes mentioned above.

Subjects experiencing adverse postoperative events and those who had an uneventful postoperative course were compared, taking into account the antral CSA, both measured and calculated, in supine and RLD positions.

Mean CSA_calculated_ in RLD was significantly higher in patients who showed these adverse events with respect to the second group (12.95 cm^2^ vs 6.12 cm^2^; *p* = 0.040).

The algorithm used by P. Van de Putte and A. Perlas [21] to predict the risk of aspiration (based on antral area as a predictor of gastric volume and on qualitative assessment of gastric contents) was applied (Fig. 4).

Predicted gastric volumes > 1.5 mL/kg and/or scans showing liquid content in both positions (i.e. Grade 2 according to the antral grading system by Perlas [10]) or solid contents were regarded as high-risk subjects.

The Van de Putte and Perlas’ algorithm managed to predict only 1 patient to be at increased risk of adverse outcomes, out of the 6 subjects who actually showed such outcomes, with a prediction rate of 16.7%.

**Fig. 6:**
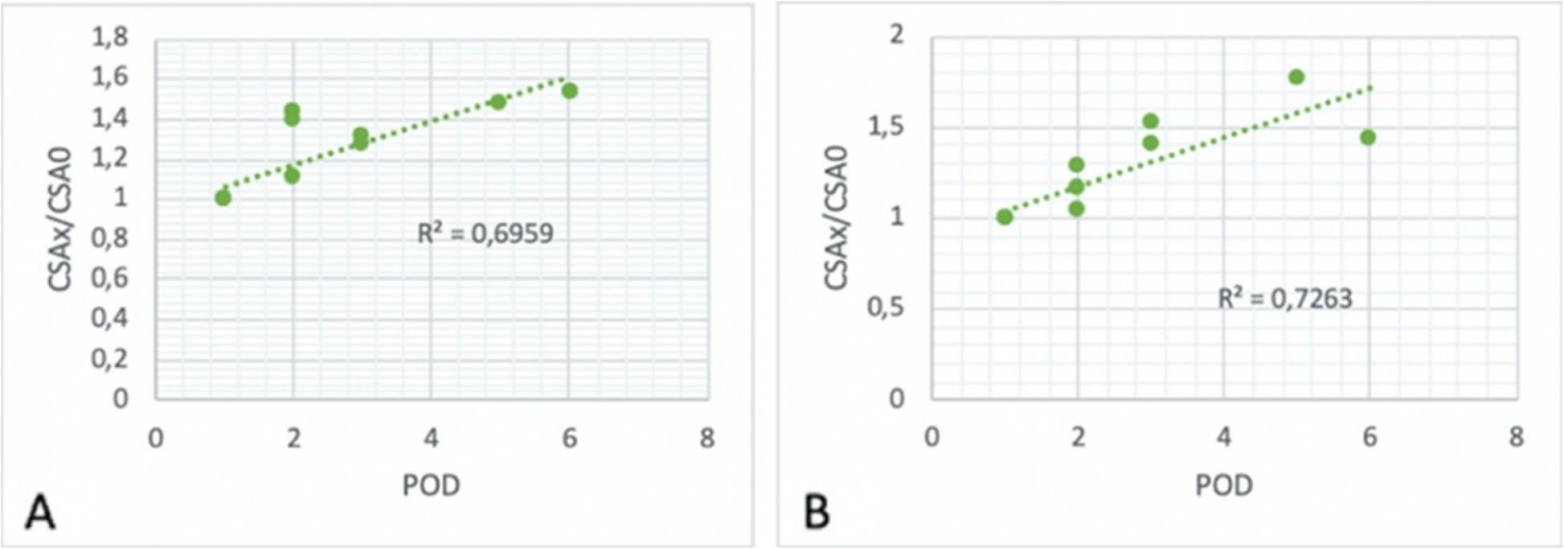
Dispersion graph with tendency line comparing post-operative day and CSAx/CSA0 in RLD (**A** CSA calculated; **B** CSA measured). CSA = cross-sectional area; POD = postoperative day

On the other hand, the measurements of patients who experienced adverse events were used to build a linear regression model which showed a good correlation between the relative variation of CSA expressed as CSA_x_/CSA_0_ [where CSA_0_ refers to the measure of CSA on the first post-operative day (POD)] and the POD in which the scan took place. This model gave out an *R*^2^ = 0.6959 for CSA_calculated_ and an *R*^2^ = 0.7263 for CSA_measured_, both in RLD position (Fig. 6).

## Discussion

US-based evaluation of gastric volume to predict PONV is a promising tool with only few published studies available. Our data are added to two other similar experiences with surgical patients.

Dioscoridi et al. [9] set an ultrasound evaluation of gastric pool using two ultrasonographic projections in 10 healthy volunteers and 20 operated patients. Results showed that gastric pool is well visible and quantifiable. The method is simple and reproducible at the bedside. The study concluded that the gastric pool’s assessment using ultrasounds is possible and useful in surgical patients in order to give indications for NGT positioning.

Jaronczyn and colleagues [11] also set a pilot study and tried to correlate the gastric distension to PONV in 20 patients who underwent colorectal surgery. The results showed that, although user dependent, US for gastric measurement can provide a means to differentiate nausea related to gastric distension from other etiologies. If gastric distension is diagnosed, then a NGT can be inserted to decompress the stomach thereby preventing vomiting and aspiration.

In our study group, overall gastric antrum detection rate was fairly high. As a matter of fact, it was possible to measure the antral CSA in about two thirds of the cases. Patient position and time of evaluation did not change detection rate significantly, but the surgical access (as well as the presence of previous laparotomy scars) did. US evaluation was feasible in almost 80% of patients who had gone through laparoscopic surgery, while detection rate went down to about 30% in patients who had undergone open surgery.

Likely, the presence of fresh surgical wounds and scars generates acoustic shadowing, which impairs US waves penetration in tissues thus decreasing technique sensitivity. Indeed, sensitivity of this technique seems variable and strongly dependent on surgical technique, presence of scars or fresh surgical wounds, that may influence US wave penetration depth. Good clinical judgement and expertise should guide the surgeon in carefully selecting patients who may benefit from this tool, and surgical incision may be limited as much as possible (e.g. by using infraumbilical mini-laparotomic incisions) in order to improve the technique detection rate and its efficiency.

Subgroup analysis shows that a dilated gastric antrum is significantly related to postoperative adverse outcomes. Thus, a careful ultrasound follow-up might help tailor postoperative nutrition and antiemetic therapy, contributing to build a proactive approach to prevention of vomiting and its complications. Our data support the possibility of implementing this non-invasive tool in specific types of surgery, where the risk of POI is at its highest.

After analyzing the data concerning the rate of postoperative events, the algorithm proposed by by Perlas and Van de Putte [21] in a review on this topic was applied.

Such algorithm is currently used in the anesthesiology field in order to predict the risk of aspiration preoperatively, and it is devised in such a way that quantitative and qualitative evaluations are integrated in order to predict which patients are at risk of aspiration.

This method was applied on this study population to enquire whether it would work on surgical patients at risk of POI.

The algorithm managed to predict the adverse events only in 1 out of 6 patients who actually had a complicated postoperative course. Thus, our results show that such algorithm is not suitable for this purpose.

Being the antral CSA strongly dependent on the individual anatomy of each subject, and given the results of the linear regression model, a relative measure should be used to predict the risk of POI. In patients who experienced adverse events, CSA showed an average increase of more than 50% over a period of 72 h after surgery, so this relative threshold could be further investigated in a future trial.

This study has some limitations. The sample size used for the regression model is relatively small and a much larger study population should be used in order to assess the utility of a relative threshold to predict POI.

Furthermore, recruitment turned out to be problematic owing to the target population of the study originating from an emergency setting.

As such, emergency abdominal interventions are never scheduled in advance, so that patient enrolment before the surgery was often difficult and 12/41 patients were evaluated postoperatively only. A more systematic implementation of this technique in the everyday clinical practice would require increasing the number of bedside US trained operators and improve the communication among them and the on-call surgical team.

## Conclusions

Gastric US (GUS) is an effective diagnostic tool in the context of emergency abdominal surgery, and a useful integration to ERAS® protocol. Our data support the implementation of gastric US into daily clinical practice in the surgical ward, in order to tailor postoperative interventions and favor an earlier recovery. In spite of this, further studies are required in order to build a standardized method and a threshold value for gastric distension, over which the placement of a NGT and administration antiemetic therapy is beneficial.

The relative CSA increase observed in our population could be assessed in the future, in a prospective-randomized trial, on a larger patient pool and with a higher number of experienced operators, investigating the impact of this decision-making model based on US measurements on harder outcomes, like length of stay or reoperation rate.

## Data Availability

The data sets used during the current study are available from the corresponding author on reasonable request.

## Abbreviations

CSA: Cross-sectional area
ERAS®: Enhanced Recovery After Surgery
ES: Emergency Surgery
GER: Gastric emptying rate
GERD: Gastro-esophageal Reflux Disease
GI: Gastro-intestinal
GUS: Gastric UltraSound
NGT: Naso-Gastric Tube
PONV: Post-operative nausea and vomiting
POI: Post-Operative Ileus
PA: Pulmonary aspiration
RLD: Right lateral decubitus
STROBE: Strengthening the Reporting of Observational Studies in Epidemiology
US: UltraSound
